# Single-cell transcriptome sequencing: recent advances and remaining challenges

**DOI:** 10.12688/f1000research.7223.1

**Published:** 2016-02-17

**Authors:** Serena Liu, Cole Trapnell

**Affiliations:** 1 Department of Genome Sciences, University of Washington, Seattle, WA, USA

**Keywords:** Single-cell RNA-sequencing, single-cell transcriptomic profiling

## Abstract

Single-cell RNA-sequencing methods are now robust and economically practical and are becoming a powerful tool for high-throughput, high-resolution transcriptomic analysis of cell states and dynamics. Single-cell approaches circumvent the averaging artifacts associated with traditional bulk population data, yielding new insights into the cellular diversity underlying superficially homogeneous populations. Thus far, single-cell RNA-sequencing has already shown great effectiveness in unraveling complex cell populations, reconstructing developmental trajectories, and modeling transcriptional dynamics. Ongoing technical improvements to single-cell RNA-sequencing throughput and sensitivity, the development of more sophisticated analytical frameworks for single-cell data, and an increasing array of complementary single-cell assays all promise to expand the usefulness and potential applications of single-cell transcriptomic profiling.

## Introduction

The advent of next-generation sequencing over a decade ago spurred the development of a host of sequencing-based technologies ^1^ for probing genomic variation and dynamics. Of these methods, RNA-sequencing (RNA-seq) enabled transcriptomic profiling at unprecedented sensitivity and breadth, leading to the discovery of new RNA species and deepening our understanding of transcriptome dynamics ^2, 3^. In recent years, low-input RNA-seq methods have been adapted to work in single cells ^4^. These single-cell RNA-seq (scRNA-seq) technologies can quantify intra-population heterogeneity and enable study of cell states and transitions at very high resolution, potentially revealing cell subtypes or gene expression dynamics that are masked in bulk, population-averaged measurements ^5, 6^. In this review, we will discuss recent advancements and current limitations of scRNA-seq methodologies and highlight major applications of scRNA-seq in biological research.

## scRNA-seq technologies: overview and recent advancements

Over the past six years, numerous scRNA-seq protocols have been developed ^4, 7– 21^. Currently published scRNA-seq protocols all follow the same general workflow: single cells are isolated; cells are lysed, and the RNA is captured for reverse transcription into cDNA; and the cDNA is pre-amplified and then used to prepare libraries for sequencing and downstream analysis. Kolodziejczyk *et al.* ^22^ provide a comprehensive review of individual scRNA-seq protocols and their relative strengths and weaknesses.

Although cDNA pre-amplification is necessary because only minute amounts of RNA are captured from each cell ^23^, amplification bias arising during pre-amplification limits the quantitative accuracy of scRNA-seq. Unique molecular identifiers (UMIs) can be used to barcode individual RNA molecules during the reverse transcription step, allowing direct transcript counting ^24– 29^, and many of the newer scRNA-seq protocols use UMIs to improve transcript quantitation ^9, 16– 19^. Alternatively, exogenous RNA standards such as those from the External RNA Control Consortium (ERCC) can be “spiked in” with cellular RNA to map between relative and absolute transcript counts ^20, 30^. Stegle *et al.* ^31^ provide a more detailed discussion of methods for scRNA-seq transcript quantitation and highlight some of the analytical challenges unique to single-cell data.

scRNA-seq methods have also been improving in terms of throughput and scalability. Whereas most earlier methods have been limited to measuring hundreds or thousands of cells at a time, recent advancements in microwell ^17^ and droplet-based ^18, 19^ cell-barcoding strategies have enabled the analysis of tens of thousands of cells in a single experiment. The high-throughput capacity of these new technologies will increase the resolution of single-cell experiments, improving their ability to detect rare cell subtypes or transitional states.

## Challenges and limitations of scRNA-seq

Current scRNA-seq technologies still face a number of challenges. Collectively, existing scRNA-seq methods have low capture efficiency. Because only a small fraction of each cell’s transcript complement (approximately 10% for many protocols ^9^) is represented in the final sequencing libraries, scRNA-seq has limited sensitivity and is unable to reliably detect low-abundance transcripts ^9, 32, 33^. The low amount of input material for scRNA-seq libraries also leads to high levels of technical noise, which complicates data analysis and can mask underlying biological variation ^22, 34– 37^. Methods for modeling technical variation in scRNA-seq data have been proposed ^35– 37^; however, most approaches use the sample-to-sample variation in ERCC read counts to model and control for technical noise in the single-cell data and thus can be used only with experiments incorporating spike-in controls. Moreover, these approaches assume that the spike-in transcripts are treated the same as cellular RNA during library prep. However, naked spike-in RNA does not pass through cellular lysis and is not in complex with ribosomes or RNA-binding proteins. Thus, although spike-in procedures serve as useful indicators of transcript frequency and sensitivity in an experiment, there are many sources of variability that remain difficult to control in scRNA-seq.

Another potential source of bias stems from procedures to isolate and capture individual cells. Although micromanipulation or laser dissection techniques can isolate single cells from known locations within a cell population or tissue, these methods are labor-intensive or require specialized equipment ^22, 33, 38^. Most scRNA-seq protocols—and all of the existing high-throughput methods—first dissociate tissues to form a single-cell suspension before capturing individual cells. This cell dissociation step is often non-trivial, and enzymatic treatments used to break down tissues may impact cell viability, potentially affecting cells’ transcriptional profiles ^22^. To avoid biases stemming from such enzymatic treatments, Grindberg *et al.* have developed techniques for performing RNA-seq directly on single nuclei ^39, 40^, which can be isolated without using harsh protease treatments.

For most single-cell isolation procedures, information about cells’ original spatial context and cellular environment is lost. Recently, computational methods have been developed to infer a cell’s original position in three-dimensional space from its transcriptional profile by using a reference gene expression map built from existing *in situ* data ^41, 42^. However, these methods rely on the existence of spatial expression data for a panel of reference genes in the tissue of interest. Alternatively, emerging *in situ* sequencing strategies are able to capture and amplify RNA within the original tissue context, although current methods can measure up to only a few dozen genes per cell ^43– 45^. These methods sequence RNA directly inside unlysed cells: cDNA amplicons are generated and circularized, amplified via rolling circle amplification, and then sequenced by ligation *in situ* by using the SOLiD platform ^44, 45^. Such *in situ* sequencing approaches are distinct from fluorescence *in situ* hybridization (FISH) strategies (discussed further below), which detect transcripts through the binding of fluorescently labeled probes. However, although *in situ* sequencing methods preserve spatial information and can measure RNA expression patterns at subcellular resolution, these approaches are currently limited in throughput and require specialized tools which may not be widely accessible.

Finally, the bulk of scRNA-seq literature has focused solely on polyadenylated mRNAs; almost all published scRNA-seq protocols isolate cellular RNA by using poly-T priming, which captures only polyadenylated transcripts. Consequently, current methods are ill suited to investigate non-polyadenylated transcript classes, such as regulatory non-coding RNA (e.g. microRNAs ^46, 47^, lncRNAs ^48^, or circular RNAs ^49, 50^) or bacterial RNA ^21^. Random hexamer priming has been suggested as a strategy to simultaneously capture both polyadenylated and non-polyadenylated transcripts in single cells ^20, 21^, and computationally selected “not-so-random” primers could potentially be used to capture poly(A)+ and poly(A)– species while depleting for ribosomal RNA ^51^. Incorporating these alternative priming strategies into existing scRNA-seq technologies would enable the exploration of a wider spectrum of transcript types, broadening the scope and applicability of scRNA-seq.

## Complementary single-cell technologies

Although scRNA-seq alone is a powerful tool for dissecting cell populations and processes, combining scRNA-seq with other single-cell technologies supplements transcriptomic data with complementary information that helps to paint a more complete picture of each cell. RNA FISH, in which individual transcripts are labeled with fluorescent probes and then detected via high-resolution microscopy, provides an orthogonal method of quantifying transcript levels and is often used to independently validate results from scRNA-seq data ^52^. Unlike scRNA-seq, single-cell FISH preserves the spatial context of assayed transcripts and can localize molecules down to subcellular resolution ^53, 54^. RNA localization and trafficking dynamics often play a crucial role in regulating protein translation and cellular function ^55^; used in conjunction with scRNA-seq, single-cell FISH could supplement the global transcriptomic snapshots of scRNA-seq with information on the spatial dynamics of selected transcripts. Whereas spectral overlap between fluorophores still limits the number of transcripts that can be simultaneously assayed, new approaches using super-resolution microscopy and combinatorial labeling schemes can measure up to thousands of transcripts in each cell ^53, 54, 56^.

Single-cell genome sequencing has been developing alongside scRNA-seq and has been used successfully to map genetic variation at single-cell resolution and to infer cell lineages ^57– 61^. Moreover, in the past year, methods have been developed to sequence both the genome and the transcriptome of the same cell ^62, 63^, enabling direct comparison of genetic and gene expression variation within a single cell. This integrated, parallel-sequencing approach shows great promise for uncovering genotype-phenotype relationships and has already been used to demonstrate strong correlations between gene copy number and gene expression levels ^62, 63^.

Over the past few years, methods have also been developed to assay the epigenetic landscape of single cells: both bisulfite sequencing ^64– 67^ (measuring DNA methylation) and assay for transposase-accessible chromatin with high-throughput sequencing (ATAC-seq) ^68, 69^ (measuring chromatin accessibility) have been adapted to work with single cells. These methods offer insight into the epigenetic heterogeneity within cell populations, and paired epigenomic and transcriptomic data could deepen our understanding of the mechanisms underlying gene expression regulation. Although direct comparison of a cell’s epigenomic and transcriptomic profiles is not currently possible, combining single-cell bisulfite sequencing or single-cell ATAC-seq with scRNA-seq from the same cell could enable such analyses in the future. Similarly, integrating scRNA-seq with single-cell proteomic methods ^70, 71^ would provide insight into post-transcriptional gene regulation and the degree to which mRNA expression is reflected at the protein level.

## Applications of scRNA-seq

Recent studies have demonstrated high cell-to-cell transcriptomic variation ^10, 72– 74^, even within genetically homogenous cell populations ^75^. Consequently, bulk measurements can mask important cellular heterogeneity ^5, 76^ and lead to averaging artifacts ^6^. One major advantage of scRNA-seq is its ability to detect such cell-to-cell heterogeneity and capitalize upon it to uncover population structure and cell dynamics hidden at the group level.

**Figure 1.**
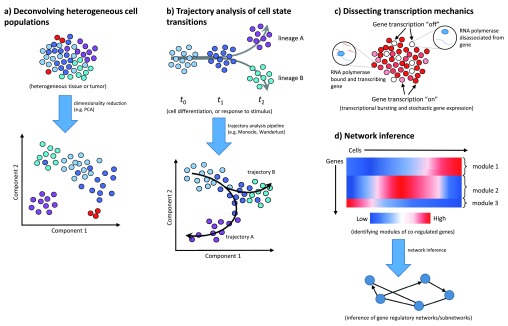
Common applications of single-cell RNA sequencing. ( **a**) Deconvolving heterogeneous cell populations. Clustering by single-cell transcriptomic profiles can reveal population substructure and enable the identification of cell subtypes and rare cell species (e.g. red cells above). Clusters may be tight and well defined (purple, red) or diffuse (blue). ( **b**) Trajectory analysis of cell state transitions. Single-cell RNA sequencing time-series data can be used to map cell developmental trajectories over the course of dynamic processes such as differentiation or signaling responses to an external stimulus. Some computational suites (e.g. Monocle ^6^) can also accommodate branching trajectories, enabling identification of lineage-specific gene expression and key genes that drive branching events. ( **c**) Dissecting transcription mechanics. Genes’ expression profiles across many cells can be compared to study transcriptional bursting and to model the kinetics of stochastic gene expression. ( **d**) Network inference. Genes can be clustered by expression profile to identify modules of putatively co-regulated genes, and gene-gene covariation relationships can be used to infer gene regulatory networks or subnetworks.

scRNA-seq has been used to dissect heterogeneous cell populations and complex tissues, such as intestine ^77^, spleen ^16^, lung ^78^, or brain ^42, 79– 83^. Clustering methods ^16, 75, 77^ or dimensionality reduction techniques ^78^ can be used directly on single-cell expression data to group cells by transcriptomic similarity and to detect the underlying population structure in an unsupervised manner ( Figure 1A). Cell subgroups identified from such analyses can often be matched to known cell types via previously established marker genes ^16, 52, 78, 81, 82^; however, structural analysis of single-cell data has also led to the discovery of novel cell subtypes ^79, 83, 84^ as well as the identification of new marker genes for known cell types ^78, 84, 85^. In the context of cancer, scRNA-seq analyses have been used to characterize intra-tumoral heterogeneity and to classify tumor subpopulations ^86– 88^. scRNA-seq profiling can also detect variation among cell states within a seemingly homogenous population, such as differences in cell cycle stage ^89^ or differential signaling responses to an outside stimulus ^52, 75, 90^.

scRNA-seq is also commonly used to study cellular transitions between different states and to map cell trajectories through processes like differentiation ( Figure 1B). Several analytical frameworks have been proposed for inferring such trajectories: Monocle introduced the concept of “pseudotime” as a quantitative measure of “progress through a biological process” and uses techniques from computational geometry to order cells in pseudotime on the basis of their transcriptomic profiles ^6^. Wanderlust uses an entirely different algorithm based on local topological clustering to place cells along a developmental trajectory ^91^ by using single-cell proteomic measurements. More recently, Shin *et al.* ^92^ and Moignard *et al.* ^93^ have outlined additional strategies for reconstructing cell trajectories. Once cells have been ordered along a trajectory, gene expression patterns over the course of the established developmental trajectory can be analyzed to identify key regulators and genes with “switch-like” behavior ^6, 72, 91^. Sensitivity for identifying intermediate differentiation states can also be improved by using latent variable models to account for potential confounding factors (such as cell cycle) in the expression data prior to applying trajectory analysis techniques ^94^.

Growing evidence suggests that genes are not transcribed continuously but rather undergo short bursts of transcription interspersed with silent intervals ^95^. Transitions between “on” and “off” states are governed by several stochastic processes ^96, 97^, and this phenomenon of “transcriptional bursting” is a major source of gene expression heterogeneity between cells. scRNA-seq can be used to explore transcriptional mechanics and to model the kinetics of stochastic gene transcription ^96, 98, 99^ ( Figure 1C). Recent studies have also reported instances of cells preferentially expressing a single allele ^32^ or a single splice isoform ^75^; however, the low mRNA capture efficiency of scRNA-seq makes it difficult to draw definitive conclusions about allele-specific or isoform-specific expression at the single-cell level.

The inherent gene expression variability between cells in scRNA-seq data can be used to infer gene regulatory networks (GRNs) ^100– 102^. Most commonly, genes are grouped into co-regulated “modules” on the basis of expression profile similarity ^16, 52, 75, 86, 87, 103^ ( Figure 1D). Network inference from scRNA-seq data poses several challenges. Owing to low capture efficiency and stochastic gene expression, gene dropout (where gene expression is zero in a given cell) is quite common, leading to zero-inflated expression data ^104^. Although zero-inflated distributions can be used to accommodate expected dropout ^104– 106^, such models also have a greater number of parameters and can be more difficult to fit than a simpler model, particularly when sample size is limited. As previously mentioned, scRNA-seq data are very noisy, and separating biological variation from technical noise remains a non-trivial problem ^35, 36^. Additionally, the number of model parameters to be estimated (genes and gene interactions) usually greatly exceeds the number of sample observations (cells measured), and this disparity poses challenges for parameter estimation ^107, 108^. Simplifying the model on the basis of prior knowledge or focusing on only a small subnetwork of key players may be necessary to make parameter estimation feasible ^107– 110^. Finally, experimentally validating inferred GRNs can be very difficult; whereas knocking out a single gene is relatively straightforward, disrupting interactions between two proteins or between a protein and its target sequence can be much harder, and very few hypothesized models have been rigorously tested thus far.

## Conclusions

scRNA-seq technologies have advanced significantly since their inception, improving in terms of both transcript quantitation and experimental throughput. Whereas low capture efficiency and high levels of technical noise limit the sensitivity and accuracy of scRNA-seq, more sophisticated analytical frameworks are emerging to facilitate the interpretation of scRNA-seq data ^35– 37^. Pairing single-cell transcriptomic data with spatial information ^41, 42, 54^ or orthogonal single-cell genomic assays ^62, 63, 65, 68^ also promises to provide new insights into transcriptional dynamics and the mechanisms underlying gene regulation.

scRNA-seq has been very effective at dissecting complex, heterogeneous cell populations, enabling unsupervised learning of population structure and the discovery of novel subtypes and rare cell species ^79, 84^. In the context of dynamic processes, cell trajectories reconstructed from single-cell transcriptomic data have provided insight into transient intermediate cell states and have helped to identify key regulator genes ^6, 91^. Finally, scRNA-seq also shows great potential for elucidating stochastic transcriptional kinetics and inferring gene regulatory networks. However, network inference from scRNA-seq data is computationally challenging and difficult to validate; inferred network models should thus be critically evaluated and experimentally tested where possible.
